# Efficacy of comprehensive group-based education in lowering body weight, uric acid levels, and diuretic use in patients with chronic kidney disease: a retrospective study

**DOI:** 10.1186/s12882-023-03293-0

**Published:** 2023-09-14

**Authors:** Azumi Hotta, Hirotsugu Iwatani

**Affiliations:** 1 Department of Nursing, National Hospital Organization Osaka National Hospital, 2-1-14, Hoenzaka, Chuo-ku, Osaka, 540-0006 Japan; 2 Department of Nephrology, National Hospital Organization Osaka National Hospital, 2-1-14, Hoenzaka, Chuo-ku, Osaka, 540-0006 Japan

**Keywords:** Group-based education, Salt restriction, Dietary therapy, Uric acid, Body weight

## Abstract

**Background:**

Patient education for the management of chronic kidney disease (CKD) is attracting attention. Therefore, this study aimed to analyze changes in body weight, uric acid, and estimated-glomerular filtration rate (eGFR) in patients with CKD after a group-based education during admission.

**Methods:**

Overall, 157 patients with CKD, who were discharged from the nephrology department of our hospital between January 2015 and October 2019, received group-based education or individual-based education by nurses at admission. Deltas of body weight, uric acid, and eGFR, 6 months from baseline, were compared between group- and individual-based education using the Wilcoxon rank sum test.

**Results:**

In total, 60 patients receiving group-based education (G group, *n =*35) or individual-based education (I group, *n =*25) during admission were included in this retrospective study. The patient characteristics at baseline were as follows: age mean, 72 ± SD 9; 16 females and 44 males; body weight, 62 ± 17 kg; eGFR median, 21 (IQR: 14, 29) mL/min/1.73 m^2^; UA, 7 (6.1, 7.5) mg/dL; and estimated intake of salt 6.9 (6.2, 8.4) g/day. Delta eGFR (mL/min/1.73 m^2^) was -1 (-3, 3) for G group and -1 (-2.5, 2) for I group (*p* = 0.8039). Delta body weight (kg) was -0.4 (-1.6, 0) for G group and 0 (-0.45, 0.95) for I group (*p* = 0.0597). Delta uric acid (mg/dL) was -1.1 (-1.6, 0.1) for G group and -0.2 (-1.1, 0.5) for I group (*p* = 0.0567).

In patients with higher sodium intake (≥ 117.4 mEq/day), delta body weight was significantly lower in the group-based education group than in the individual-based education group (*p* = 0.0398).

**Conclusions:**

A comprehensive group-based education in patients with CKD may effectively suppress body weight and uric acid in 6 months along with less frequent diuretic use.

## Background

The kidney manages our body’s fluid level. Therefore, monitored dietary salt intake is crucial in the management of kidney diseases. As patients with reduced glomerular filtration rate (GFR) experience a decreased ability to excrete salt, excessive intake of salt leads to body weight gain. Poor control of body fluid in patients with chronic kidney disease (CKD) often leads to the increased use of diuretics, which often accompanies hyperuricemia as an adverse effect. Moreover, the excessive intake of salt upregulates blood pressure. This hypertensive state finally leads to an increased risk for cardiovascular and end-stage renal disease [1–3].

Patient education/patient care is imperative for the long-term treatment of CKD, which includes pharmacotherapy such as the use of antihypertensives, including ACE inhibitors or ARB, along with diuretics as well as a diet encompassing salt restriction. Diets are enormously impacted by the patient’s likes and dislikes, which are to be implemented three times a day for the entirety of the patient’s life, wherein self-management and self-discipline play a crucial role in sustaining the required lifestyle modification. Keeping the patient highly motivated is the key to sustenance. To strengthen the patient’s self-management, the physician-prescribed medication or explanation about the disease in a short time at regular visits would not suffice. Here, patient education and patient care by the nursing staff apart from the physician may also contribute. Recently, several reports of patient education/patient care in CKD management indicate a favorable outcome [4, 5]. For example, a study previously reported the albuminuria-lowering effect of intensive low-salt diet education [6]. Additionally, this education may be enhanced by providing it in a group setting [7].

However, there have been few reports on the association of group-based education and body weight, uric acid, or diuretic use in patients who receive it in combination with the standard therapy. In this retrospective study targeting patients with CKD, we aimed to analyze how group-based education provided to patients with CKD during their hospital stay affects the status of body weight, uric acid, eGFR, or diuretic use in patients following discharge compared to individual-based education.

## Methods

We aimed to retrospectively evaluate the impact of 6 months of group-based patient education for the management of CKD. We targeted inpatients with CKD having an optimal disease condition in the nephrology department. Therefore, we set day 0 as the day immediately before discharge, with blood and urine analysis on the same day.

**Fig. 1:**
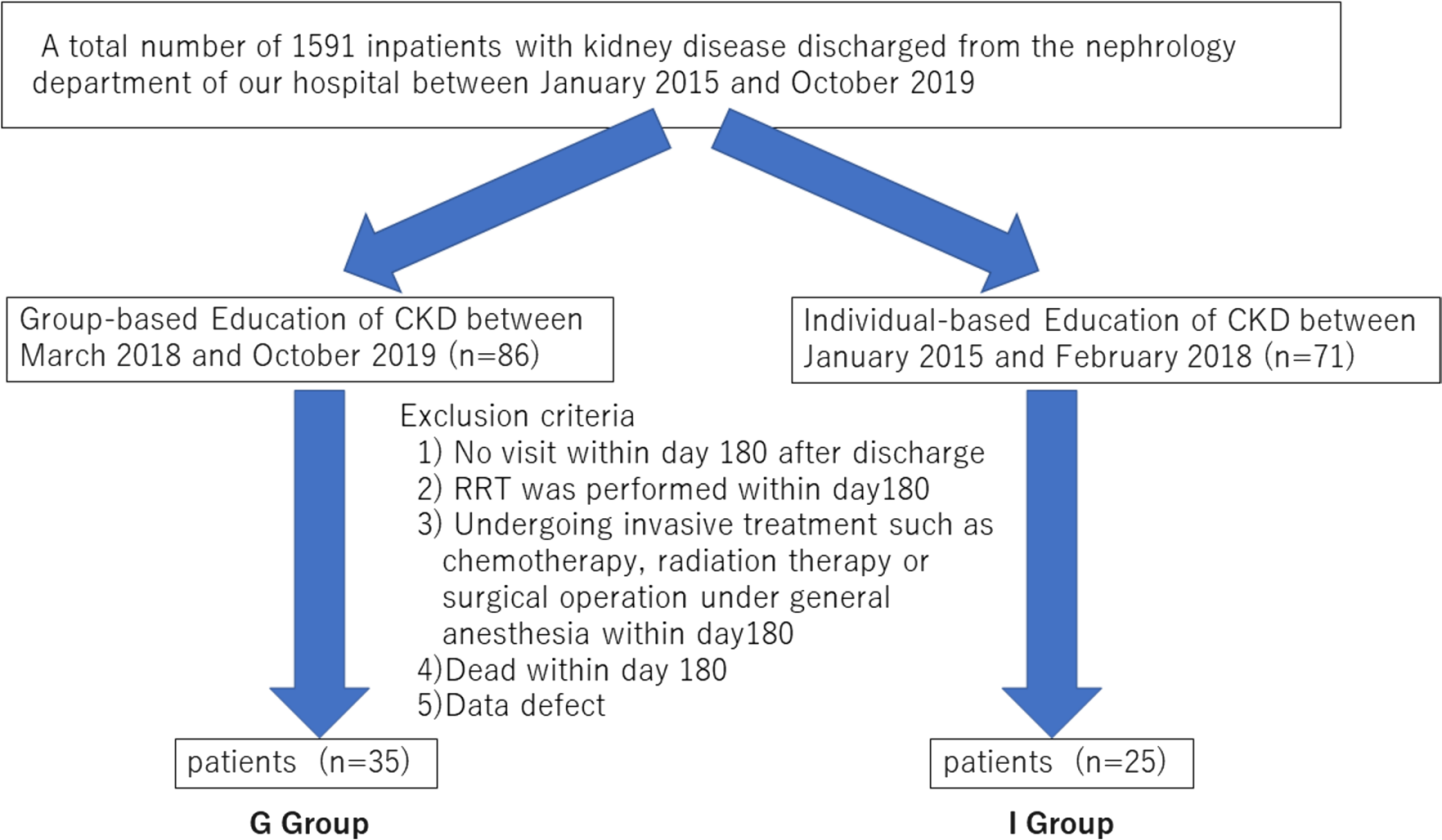
Flowchart of the patient recruitment. Abbreviations: CKD, chronic kidney disease; RRT, renal replacement therapy

Among 1591 inpatients with kidney disease discharged from the nephrology department of our hospital between January 2015 and October 2019, 157 patients obtained group-based education (*n =*86) or individual-based education (*n =*71) by nurses during admission. The following exclusion criteria were applied to 157 patients: 1) no visit within day 180 after discharge; 2) renal replacement therapy performed within day 180 after discharge; 3) undergoing invasive treatment such as chemotherapy, radiation therapy, or surgical operation under general anesthesia within day 180 after discharge; 4) death within day 180 after discharge; or 5) data defect. Ultimately, 60 patients were included as the participants of the present study. The patients who underwent group-based education for CKD as inpatients from March 2018 to October 28, 2019 (*n =*35, group-based education group), were compared with the historical control group of patients who underwent individual-based education for CKD as inpatients from January 2015 to February 2018 (*n =*25, individual-based education group). The patient enrollment flow is shown in Fig. 1.

In the group-based education category, a small number (2–4) of inpatients with CKD, occasionally along with their relatives, gathered in a room (total participants including their relatives were ≤6). A nurse with adequate knowledge and experience in CKD patient care explained (A) the mechanism of the kidney disease, (B) food and/or drink that was suitable or not for intake, (C) the interpretation of the laboratory data, and (D) the significance of the pharmacotherapy. Moreover, lifestyle modifications, such as salt restriction or low-protein diet, were a key topic concerning which food and/or drink to take or abstain from. In the group discussion, patients not only learned from the nurse, but also through a discussion with other patients. The following points were carefully considered:

1. Use a checklist that helps to roughly estimate the salt intake before admission by showing the salt content of various foods or seasoning agents (salt or soy sauce) with illustrations (https://www.ono-c.com/pdf/lecture01.pdf).
2. Use of photos or nutritional ingredient tables on food products or seasoning agents.
3. Visual emphasis by using a projector device and a big screen.
4. Appointing time for discussion for approximately 30 min. A discussion between the nurse and patients, accompanied by a discussion among patients, was incorporated.
5. The explanation of the renal function. Firstly, the symptoms of the participants with CKD, such as edema or decreased urinary volume, were referred by the nurse. The conversation about such symptoms by the participants was helpful for the participants who do not experience such symptoms to understand the disease itself or its progression. This discussion was further helpful in understanding the significance of measuring the urinary volume or the water volume to drink during admission.
6. The explanation of the prescribed medicine. Most of the participants were taking several medications and asked the nurse regarding the reasons for consuming these medicines. The nurse elucidated the functions of the kidney and subsequently emphasized the corresponding medication to each function of the kidney. In this discussion, the participants who took only antihypertensives learned a lot from the other patients who were prescribed additional medications, thereby understanding the natural course of the disease progression gravely and therefore got motivated to modify their inadequate lifestyle to avoid needing more medications in the future.

For the group-based education, it took approximately 1 h to complete the education program.

In the individual-based education group, the nurse responsible for patient care for the day explained the content of (A), (B), (C), and (D) to the patient one on one. The education method was up to the nurse in charge for the day. Total content of the education was divided by several sessions performed on different days.

In general, salt restriction a slightly below than 6 g/day and mild protein restriction of 0.8 g/kg/day were encouraged both in the group-based education group and in the individual-based education group. The comparison of the topics and methodology between the group-based education group and the individual-based education group were summarized in Table 1. Approximate daily content of sodium and protein in the meal on day 0 was shown in Table 2.

**Table 1:** Comparison of the topics and methodology between G group and I group

		G group	I group
Topic	Clinical symptoms	●	●
	Function of the kidney		
	Interpretation of the laboratory data (blood, urine)	●	●
	Explanation of the prescribed medicine	●	●
	Food and/or drink that was suitable or not for intake	●	●
	Life style modifications, such as salt restriction or low-protein diet	●	●
	Measurement of blood pressure and body weight	●	●
Methodology	Number of participating patients	2 ~ 4	1
	Number of sessions	1	Several times (Total content was divided by several times)
	Information flow	Multi-directional among nurse and patients	One way from nurse to a patient
	Discussion among patients	●	None
	Checklist to roughly estimate the salt intake	●	Not determined
	Photos or nutritional ingredient tables on food products or seasoning agents	●	Not determined
	Visual emphasis by using a projector device and a big screen	●	None

*G group* Group-based education group, *I group* Individual-based education group

●:present

**Table 2:** Approximate daily content of sodium and protein in the meal on day 0

	**Total**	**G group**	**I group**
Sodium content, (equivalent to NaCL, mEq/day)	100 (100, 100)	100 (100, 100)	100 (100, 100)
protein content, g/day	45 (45, 50)	45 (45, 50)	45 (45, 50)

Data are presented as the median (interquartile range)

*G group* Group-based education group, *I group* Individual-based education group

We collected data regarding age, sex, body weight (BW), systolic blood pressure, serum creatinine, uric acid (UA), urine sodium, urine creatinine, and medication on day 0. Additionally, we obtained the height data, measured on the first day of admission.

Moreover, we collected BW, serum creatinine, UA, and medication on day 180 after discharge. The allowance of the definition of day 180 was within 180 ± 35 days.

We defined the delta (∆) of each parameter as follows.

$$\triangle\mathrm{eGFR}\;=\;\left(\mathrm{eGFR}\;\mathrm{on}\;\mathrm{day}\;180\right)\;-\;\left(\mathrm{eGFR}\;\mathrm{on}\;\mathrm{day}\;0\right)$$

$$\triangle\mathrm{BW}\;=\;\left(\mathrm{BW}\;\mathrm{on}\;\mathrm{day}\;180\right)\;-\;\left(\mathrm{BW}\;\mathrm{on}\;\mathrm{day}\;0\right)$$

$$\triangle\mathrm{UA}\;=\;\left(\mathrm{UA}\;\mathrm{on}\;\mathrm{day}\;180\right)\;-\;\left(\mathrm{UA}\;\mathrm{on}\;\mathrm{day}\;0\right)$$

We used the electric data capture system from the medical record available. To estimate the urinary sodium excretion per day, we employed the following equations: estimated urinary sodium excretion per day [mEq/day] = 21.98 * {0.1 * (spot urine sodium concentration [mEq/L])*(estimated urinary excretion of creatinine per day [mg/day])/(spot urine creatinine concentration [mg/dL])} ^0.392^ [8].

The estimated urinary excretion of creatinine per day was calculated using the following equation developed by our group: 24 h-uCr (mg/day) = [-9.04 × age (years) + 8.03 × weight (kg) + 0.66 × height (cm) + 188.59 (if male)—32.11 × serum Cr (mg/dL) + 779.14] [9].

The estimated salt intake per day [g/day] was calculated by estimated urinary sodium excretion per day [mEq/day] /17.

Statistical data are presented as mean ± standard deviation (SD) or the median (interquartile range [IQR]), depending on the distributional normality of the data. To compare the delta (∆) of each parameter between the two groups, the Wilcoxon rank sum test was adopted using JMP 8 (SAS Institute Inc., Cary, NC, USA). Analyses were considered significant when the *P* value was < 0.05.

## Results

The baseline patient characteristics are illustrated in Table 3, where all the data were measured on day 0, and height was collected from that measured on the day of admission. The difference between day 0 and the day of discharge was observed to be a median of 2 (IQR: 1, 3) days.

**Table 3:** The baseline patient characteristics on day 0

	**Total**	**G group**	**I group**
N	60	35	25
Age, y	73 (67, 78)	73 (67, 78)	71 (66, 78)
Sex F/M	16/44	12/23	4/21
Height, cm	160 ± 10	162 (152.7, 167)	161 (151.9, 168.7)
Body weight, kg	59.2 (50.7, 67.4)	57.7 (49.7, 71.5)	60.6 (54.3, 65.3)
BMI	23.3 (20.2, 25.9)	23.6 (20.0, 26.0)	22.9 (21.7, 25.6)
eGFR, ml/min/1.73m^2^	21 (14, 29)	20 (14, 34)	21 (17, 28)
Uric Acid, mg/dL	7 (6.1, 7.5)	7.2 (6.3, 7.6)	6.6 (6, 7.4)
SBP, mmHg	130 (123, 146)	129 (123, 140)	138 (122, 148)
Diabetes	28 (47)	16 (46)	12 (48)
Hypertension	56 (93)	33 (94)	23 (92)
Coronary artery disease	9 (15)	4 (11)	5 (20)
Use of diuretics	25 (42)	13 (37)	12 (48)
Use of UA-lowering medication	44 (73)	25 (71)	19 (76)
Estimated urinary Cr excretion, mg/day	785 (650, 906)	784 (589, 873)	820 (672, 913)
Estimated urinary Na excretion, mEq/day	117.4 (106.0, 142.0)	113.9 (101.1, 139.5)	118.9 (114.4, 146.2)

All the data were measured on day 0, but height was collected from that measured on the day of admission

Data are presented as the mean ± standard deviation, the median (interquartile range), or the actual number (%)

*Abbreviations*: *SBP* systolic blood pressure, *UA* Uric Acid, *G group* Group-based education group, *I group* Individual-based education group

In the total population, the median eGFR, body weight, uric acid (UA), and estimated urinary sodium excretion were 21 (IQR: 14, 29) mL/min/1.73 m^2^; 59.2 (IQR: 50.7, 67.4) kg; 7 (IQR: 6.1, 7.5) mg/dL; and 117.4 (IQR: 106.0, 142.0) mEq/day, respectively.

**Fig. 2:**
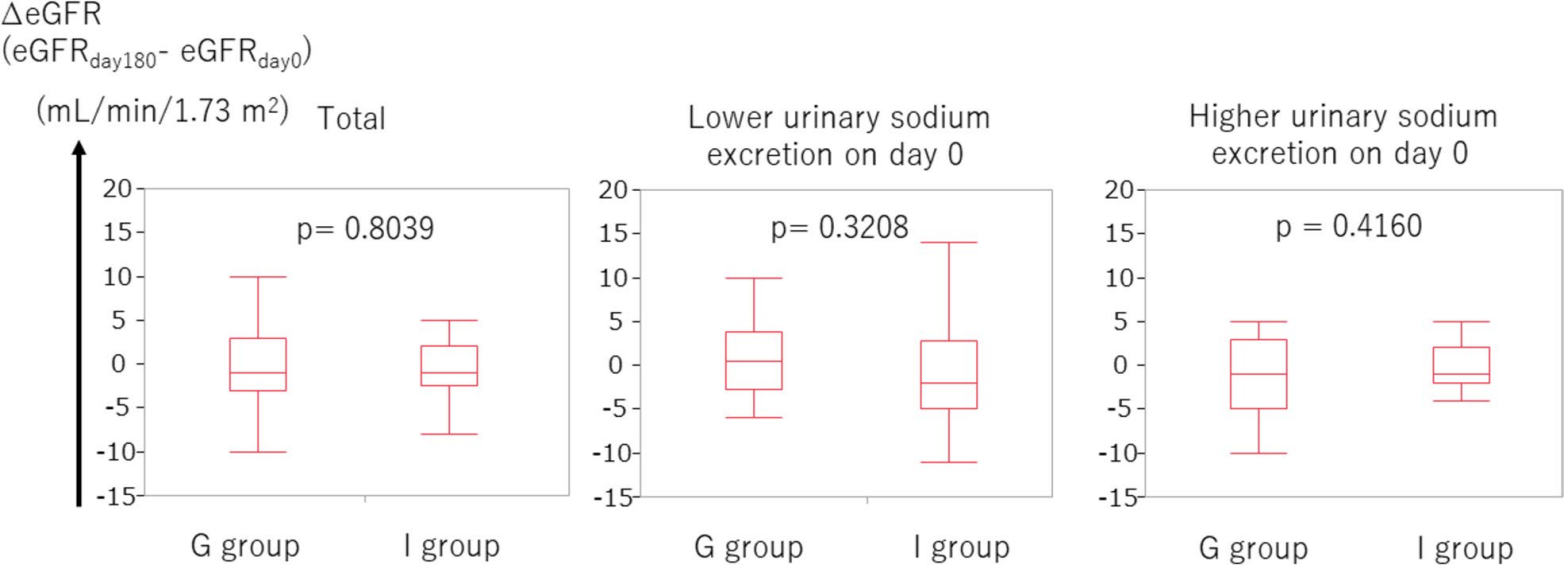
There was no statistical difference in ∆eGFR between the group-based education group and individual-based education group. Higher and lower urinary sodium excretion corresponds to urinary sodium excretion ≥ 117.4 mEq/day and < 117.4 mEq/day, respectively

The clinical parameters on day 0, day 180, and the deltas of each parameter are shown in Table 4. Firstly, we investigated the ∆eGFR, defined as (eGFR on day 180) – (eGFR on day 0). No statistical difference was found in ∆eGFR between the group-based education group and individual-based education group (*p* = 0.8039, Fig. 2).

**Table 4:** Clinical parameters on day 0 and on day 180

	**Day 0**		**Day 180**		**∆(Day 180 – Day 0)**	
	**G group**	**I group**	**G group**	**I group**	**G group**	**I group**
Body Weight, kg	57.7 (49.7, 71.5)	60.6 (54.3, 65.3)	57.5 (48.5, 70)	60.4 (55.9, 68.4)	-0.4 (-1.6, 0)	0 (-0.45, 0.95)
eGFR, ml/min/1.73m^2^	20 (14, 34)	21 (17, 28)	19 (13, 39)	20 (12, 35.5)	-1 (-3, 3)	-1 (-2.5, 2)
UA, mg/dL	7.2 (6.3, 7.6)	6.6 (6.0, 7.4)	6.0 (5.3, 6.5)	6.3 (5.7, 6.8)	-1.1 (-1.6, 0.1)	-0.2 (-1.1, 0.5)

Data are presented as the median (interquartile range)

*Abbreviations*: *UA* Uric Acid, *G group* Group-based education group, *I group* Individual-based education group

**Fig. 3:**
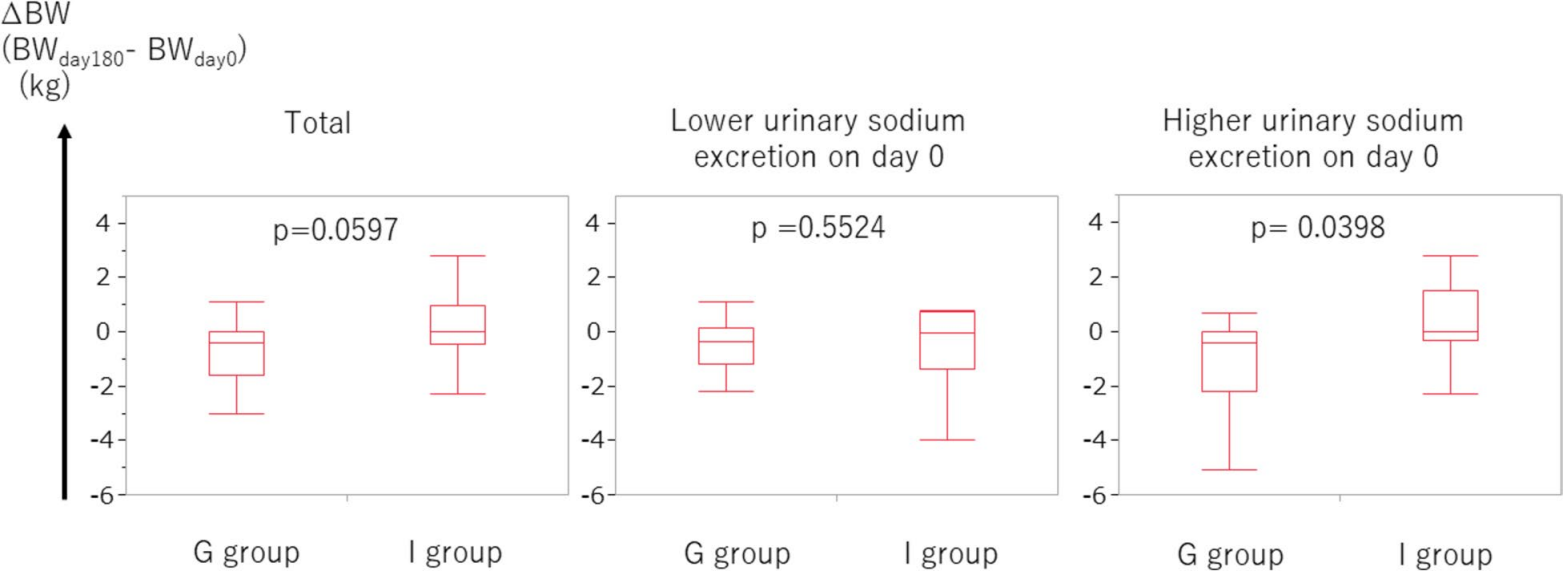
∆BW in the group-based education group tended to be lower than that in the individual-based education group. In patients with higher urinary sodium excretion, ∆BW in the group-based education group was significantly lower than that in the individual-based education group. Higher and lower urinary sodium excretion corresponds to urinary sodium excretion ≥ 117.4 mEq/day and < 117.4 mEq/day, respectively. Abbreviations: BW, body weight

**Fig. 4:**
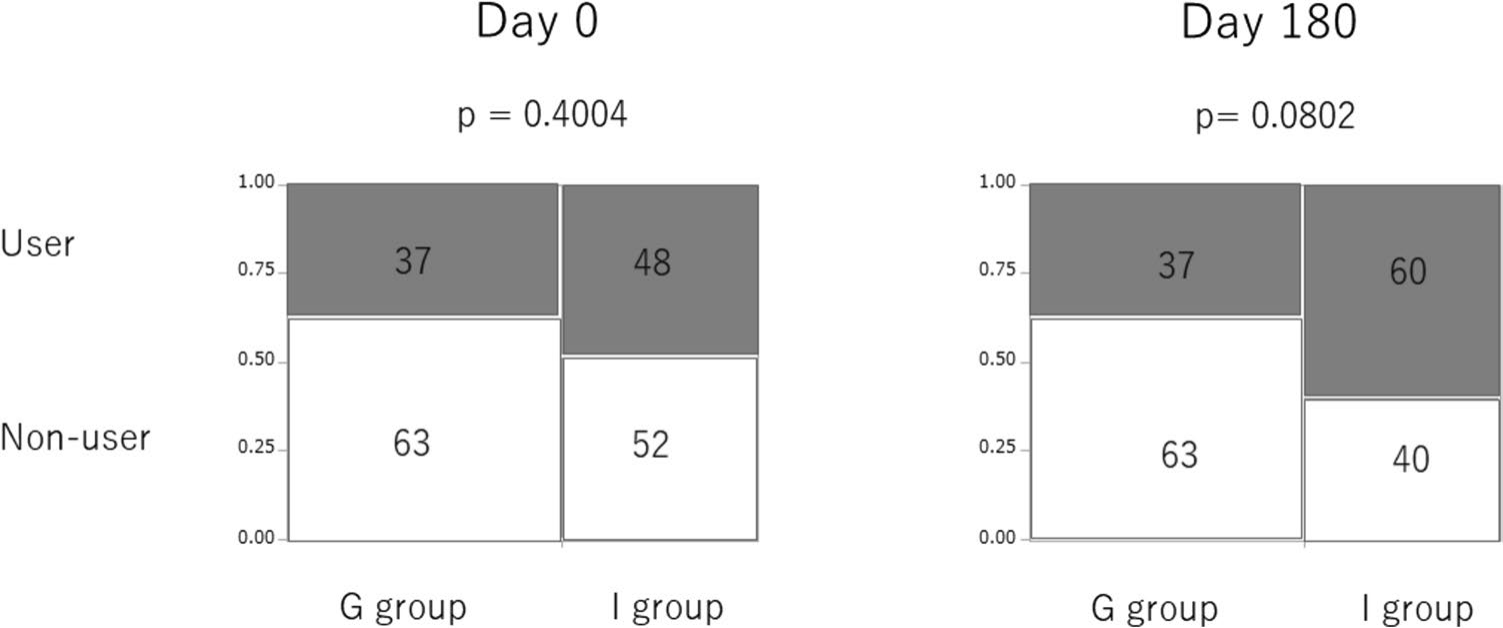
Use of diuretics. The percentages of each group are expressed in each box. User and non-user of diuretics are expressed as gray and white box, respectively

In patients with CKD, body fluid tends to extremely increase because of poor control, often leading to an increase in body weight. Secondly, we evaluated the ∆BW, which is defined as (BW on day 180) – (BW on day 0). The ∆BW in the group-based education group tended to be lower than that in the individual-based education group (*p* = 0.0597, Fig. 3). When the focus was on patients with higher urinary sodium excretion (≥ 117.4 mEq/day, corresponding to the median), the ∆BW was significantly lower in the group-based education group than in the individual-based education group (*p* = 0.0398, Fig. 3). Furthermore, we examined the use of diuretics on days 0 and 180. On day 0, there was no remarkable difference between the frequency of diuretic use between the group-based education group and individual-based group (*p* = 0.4004, Fig. 4). However, on day 180, the frequency of diuretic use in the group-based education group tended to be lower than that in the individual-based group (*p* = 0.0802, Fig. 4).

**Fig. 5:**
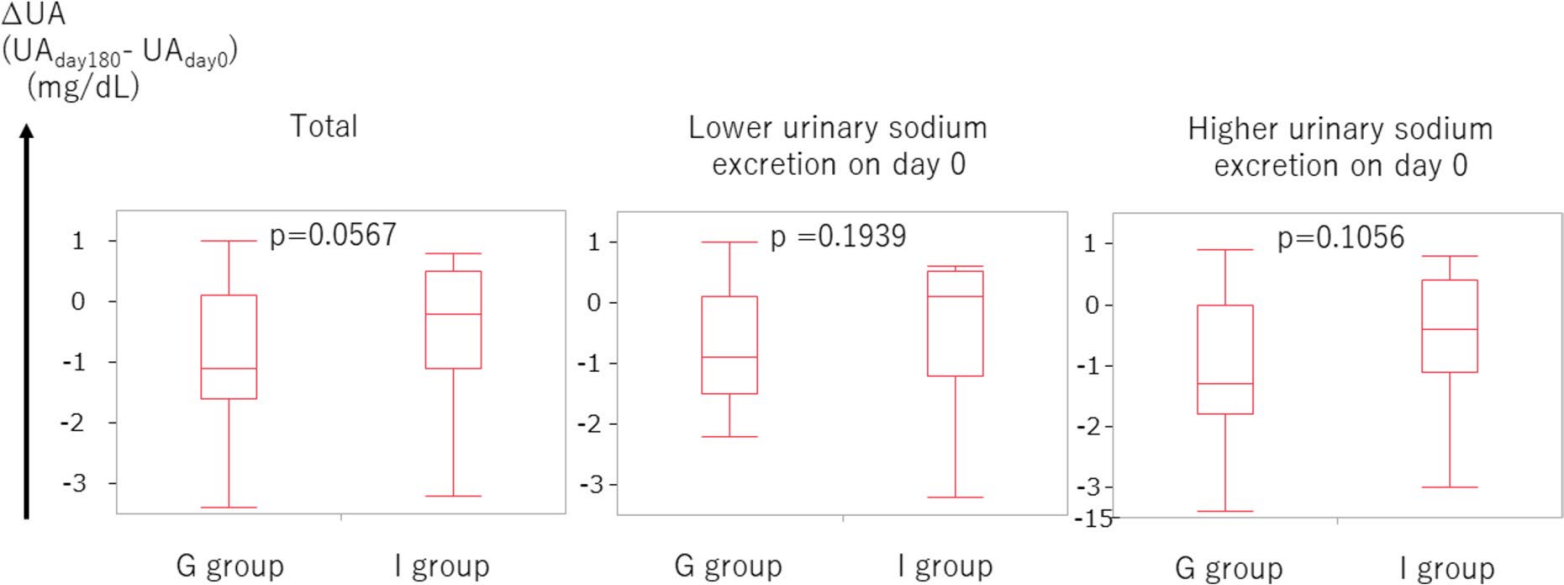
∆UA in the group-based education group tended to be lower than that in the individual-based education group Higher and lower urinary sodium excretion corresponds to urinary sodium excretion ≥ 117.4 mEq/day and < 117.4 mEq/day, respectively. Abbreviations: UA, uric acid

**Fig. 6:**
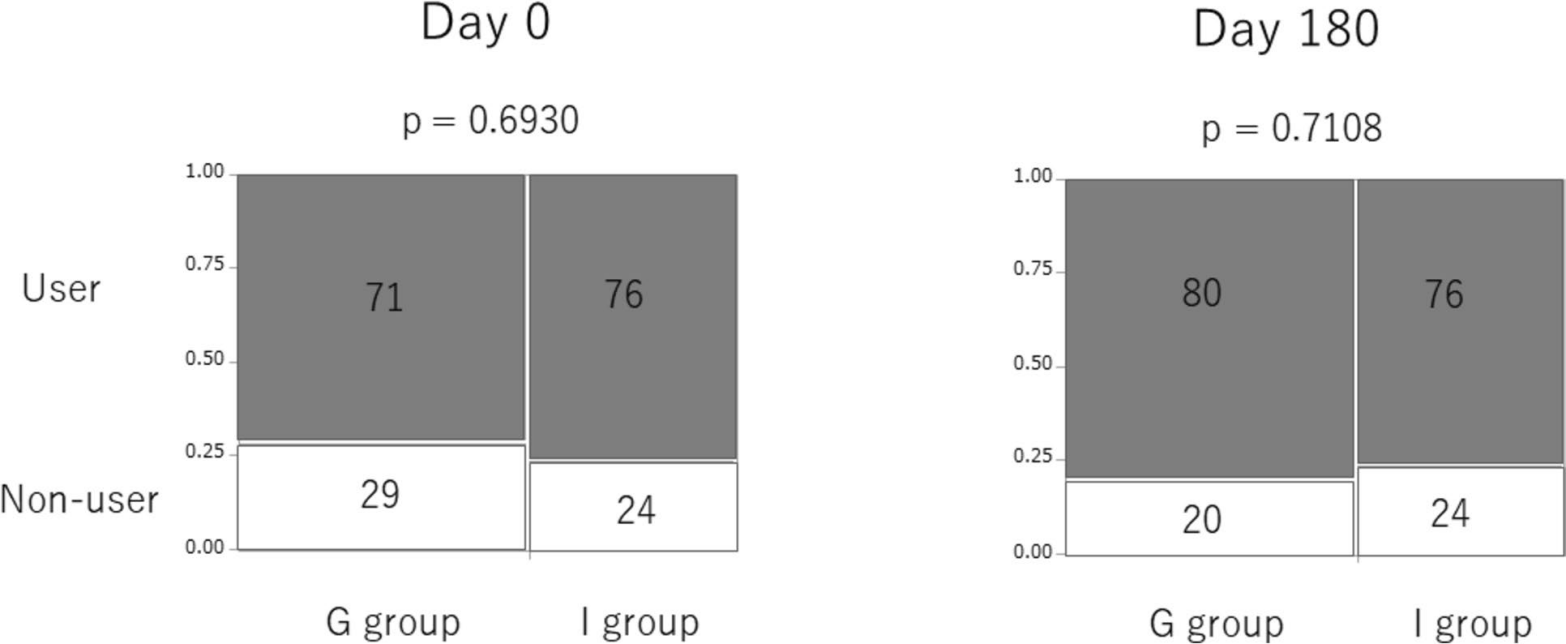
Use of UA-lowering medication. The percentages of each group are expressed in each box. User and non-user of UA-lowering medications are expressed as gray and white box, respectively. Abbreviations: UA, uric acid

Thirdly, we investigated the ∆UA, which is defined as (BW on day 180) – (BW on day 0). The ∆UA in the group-based education group tended to be lower than that in the individual-based education group (*p* = 0.0567, Fig. 5). We further assessed the use of UA-lowering medication on days 0 and 180. No statistical difference was found in the frequency of the UA-lowering medication use between the group-based education group and individual-based education group on day 0 (*p* = 0.6930, Fig. 6). This was also true on day 180 (*p* = 0.7108, Fig. 6).

## Discussion

The present study revealed three major findings. Firstly, body weight in the group-based education group decreased more than that in the individual-based group in 6 months with marginal significance. In patients with higher sodium intake, body weight in the group-based education group decreased remarkably more than in the individual-based education group in 6 months. Secondly, the use of diuretics tended to be less frequent in the group-based education group than in the individual-based education group in 6 months. Thirdly, the increase of UA in the group-based education group was less than that in the individual-based education group in 6 months with marginal significance.

Since our study intended to assess the effect of patient education and care, which may not be as effective as medication therapy or may be as easily disrupted and covered by severe disease conditions like chemotherapy, radiation therapy, or surgery, we excluded patients who underwent chemotherapy, radiation therapy, or surgery; who were unstable and required renal replacement therapy; or who died within 180 days of discharge.

Since day 0 is the day immediately before discharge, with blood and urine analysis on the same day, we can state that patients with kidney disease are generally the most stable on day 0. The group-based education involves a comprehensive education program that includes aspects such as kidney function, biochemical assays of blood and urine, nutritional education, and significance of prescribed medicine. A lifestyle modification, such as salt restriction or low-protein diet, is crucial as well. In the group-based education program, a small number of patients, including their family relatives who are the caregivers, participate in the educational program. Moreover, patients not only learned from the coaching nurse but also through a discussion with the nurse or other patients. This active learning method is completely different from the passive learning method which is often seen in an individual-based education where information is transmitted from coaching nurse to a patient in one way. Various opinions, thoughts and experiences of other patients who suffer from the same kidney disease in the group-based education could be one of the valuable learning resources other than the contents written on the textbook. That is, this active, mutual and interactive mode of an educational program would evoke a sense of togetherness and sympathy among patients, leading to a deep understanding of CKD and highly motivated participants to change their inadequate lifestyle and develop self-discipline. In the group-based education, patients may be able to perceive themselves objectively in discussing with other patients by seeing other patients, rather than subjectively, just worried and depressed by the disease progression. These would be the major reasons why our group-based education was superior to the individual-based education where the content can be tailored individually in general, which seems efficient at a glance.

This may be an advantage of a group-based education program, and some reports favor their efficacy. Narva et al. have reported that education efficacy possibly increases by providing education in group settings [7]. In addition, Johns et al. have shown that group-based care may be a promising alternative to standard nephrology care alone in adult patients with CKD [10].

No statistical difference was noted in ∆eGFR in 6 months between group-based and individual-based education groups. However, the body weight in the group-based education group decreased more than that in the individual-based education group in 6 months with marginal significance. In patients with higher sodium intake, body weight in the group-based education group notably decreased more than that in the individual-based education group in 6 months. This trend of body weight reduction in the group-based education group was attained, although there seemed to be a tendency of less frequent use of diuretics in the group-based education group than in the individual-based education group in 6 months. Furthermore, the tendency of a smaller increase of UA in the group-based education group than in the individual-based education group in 6 months was presumed to be attained by a tendency of lower use of diuretics in the group-based education group in 6 months. As hyperuricemia is a well-known adverse effect of diuretics [11, 12], the smaller increase of UA in the group-based education group is compatible with the lower use of diuretics in the group-based education group. The use of UA-lowering medication in 6 months in the group-based education group was comparable to that in the individual-based education group, which also supports the lower use of diuretics in the group-based education group.

In summary, (1) a tendency for a greater reduction of body weight, (2) a tendency of less frequent diuretic use, (3) a tendency for less increase of UA, and (4) not frequent use of UA-lowering agent in 6 months in the group-based education group than in the individual-based education group were confirmed. One possibility is that salt restriction was attained in the group-based education group in 6 months. The points (1–4) would not be attained without good adherence to adequate salt restriction in the group-based education group. When patients were focused based on higher sodium intake, the reduction of body weight was significant, which suggests that salt restriction may be attained by group-based education. In a previous report, the mean weight in the lower salt intake group was 1.32 kg lower (1.94 lower to 0.7 lower) than in the higher salt intake group [13], which also supports our results. Another possibility is that some metabolic effects have been achieved through group-based education. For example, an inverse relationship between UA levels and protection from CKD incidence and progression was reportedly observed, suggesting that lower UA levels were protective against the risk of CKD incidence and progression [14, 15]. Therefore, group-based education was associated with lowering UA and a lower risk of CKD progression. A report has shown that an increase in body weight is independently associated with an increased risk of CKD, even when the BMI is within the normal range [16]. This further indicates that group-based education was associated with lower body weight and a lower risk of CKD progression. Consequently, comprehensive group-based education for CKD patients would impact a lowered risk of CKD progression.

This study had some limitations. First, the number of participants was small, which may limit the generalizability of our results. Second, our study excluded some patients with severe conditions requiring surgical operation under general anesthesia, chemotherapy, or radiation therapy, indicating that patients with these severe conditions cannot be assessed, and our results are not applicable to such patients. Third, owing to the lack of adequate urine data on day 180, we cannot estimate the salt intake on day 180.

## Conclusions

In conclusion, comprehensive group-based education for CKD patients may effectively lower body weight and uric acid in 6 months with less frequent diuretic use, leading to a lowered risk of CKD progression. When patients were limited to those with higher sodium intake, the suppression of body weight was significant, which suggests that salt restriction may be achieved by group-based education. Considering the limitations in our study, this does not apply to patients with severe conditions requiring surgical operation under general anesthesia, chemotherapy, or radiation therapy, patients who died or patients who required renal replacement therapy in 6 months. Further prospective studies with a larger number of patients are warranted to elucidate the effect of group-based education in the future.

## Data Availability

The datasets used and/or analyzed during the current study are available from the corresponding author on reasonable request.

## Abbreviations

BW: Body weight
CKD: Chronic kidney disease
GFR: Glomerular filtration rate
IQR: Interquartile range
IRB: Institutional review board
SD: Standard deviation
UA: Uric acid
